# Influence of three artificial light sources on oviposition and half-life of the Black Soldier Fly, *Hermetia illucens* (Diptera: Stratiomyidae): Improving small-scale indoor rearing

**DOI:** 10.1371/journal.pone.0197896

**Published:** 2018-05-24

**Authors:** Carina D. Heussler, Andreas Walter, Hannes Oberkofler, Heribert Insam, Wolfgang Arthofer, Birgit C. Schlick-Steiner, Florian M. Steiner

**Affiliations:** 1 Department of Ecology, University of Innsbruck, Innsbruck, Austria; 2 Department of Microbiology, University of Innsbruck, Innsbruck, Austria; 3 Department of Biotechnology & Food Engineering - MCI - Management Center Innsbruck, Innsbruck, Austria; Universita degli Studi della Basilicata, ITALY

## Abstract

*Hermetia illucens* (L.), the Black Soldier Fly, has received increased scientific attention for its potential in circular waste management where larvae can serve as feedstuff for livestock and for biodiesel production. The flies occur naturally in (sub)-tropical and warm-temperate climates, and their mating depends on space and sunlight. Small-scale indoor rearing of Black Soldier Flies has been challenging because they react sensitive to artificial light sources and cage sizes, but recent studies have shown that small-scale rearing under artificial light is feasible. Here, we test the influence of three artificial light sources (light-emitting diodes, fluorescent lamps, and halogen lamps) on small-scale indoor rearing. Three experiments were conducted to compare oviposition traits (pre-oviposition period, total oviposition-period, and egg mass per female) and half-life among the three light sources. Oviposition did not differ among the three light sources, but male and female half-life did. Based on the performance of the light-emitting diodes and their outstanding energy efficiency, we recommend this light source for small-scale indoor rearing of Black Soldier Flies.

## Introduction

*Hermetia illucens* (L.), the Black Soldier Fly (BSF; Diptera: Stratiomyidae) [1], has raised attention due to its potential as a biological waste management agent [2]. The larvae of BSF are capable of consuming a wide range of organic residues, including animal and human faeces, urban organic waste, and plant residues [2–5]. BSF are not considered as pest insects [6]. Their larvae have the ability to reduce pathogens such as *Escherichia coli* and *Salmonella* spp. in chicken manure [7–9] and prevent the colonization by *Musca domestica* [10]. Because BSF adults have no functioning mouthparts and live on the protein and fat stored by the larvae, the latter have a high content in proteins (prepupal larvae: 40–45% of dry matter) and fat (prepupal larvae: 30–35% of dry matter) and can be used as feedstuff for livestock such as poultry, swine, and fish or can be used to produce biodiesel [11, 12]. Furthermore, BSF larvae are capable of self-harvesting as they actively migrate out of the humid substrate to dry pupation sites [13–15].

The BSF has suitable habitats in tropical, subtropical, and warm temperate regions [16]. It has optimal environmental conditions at 27 °C, 60% relative humidity (RH), and direct sunlight to successfully mate and develop [17, 18]. Eggs are deposited directly on or near a humid decomposing material and hatch approximately after four days [19]. Larvae grow and develop for approximately 14 days and turn into prepupae, and adults eclose about 14 days after pupation, depending on the environmental conditions [20]. Mating behavior of the BSF depends on light [20, 21] and is most successful under direct sunlight [19, 20].

In temperate regions, BSF are normally kept in net cages ranging from 1800 × 1200 × 1500 mm to 2000 × 2000 × 4000 mm in greenhouses under the influence of sunlight [17, 20]. However, these systems are challenging because of the cold temperatures and short days in winter. Nakamura et al. (2016) demonstrated that rearing of BSF is possible in down-scaled indoor rearing systems (270 × 270 × 270 mm) using artificial light sources for a successful and efficient indoor cultivation [22, 23].

The aim of this study was to test differences in selected oviposition traits (pre-oviposition period, total oviposition-period, and egg mass per female) and half-life of male and female adult BSF under three artificial light sources, namely, light-emitting diodes (LED), fluorescent lamps (FL), and halogen lamps (HL). The light sources were chosen to compare broadly available lamps with lamps used in previous studies. Furthermore, the light treatments were tested in small cages to validate previous studies concerning small-scale rearing [22, 23].

## Material and methods

### Breeding of Black Soldier Fly

To establish a bench-scale colony of *Hermetia illucens*, 1000 g larvae were obtained from Hermetia Baruth GmbH (Baruth, Germany). Larvae were reared in black plastic boxes (180 × 120 × 80 mm) in a Fitotron^®^ SGC 120 incubator (Weiss Technik UK, Leicestershire, United Kingdom) at 27 °C and 60% RH, following Tomberlin et al. (2009) [18]. The bottom of the boxes was covered with autoclaved (121 °C, 2 bar, 40 min.) pine humus (Dehner Terra, Rain, Germany). Following Sheppard et al. (2002), larvae were fed twice a week with 60% (w/v) ground chicken feedstuff (Grünes LegeKorn Premium, Landwirtschaftliche Genossenschaft Klagenfurt, Austria) in tap water *ad libitum* until pupation [17]. Pupae were separated manually and collected into white plastic cups (50 ml) filled with wood shavings (Dehner Terra, Rain, Germany). The cups were covered with a net (fiberglass; 150 × 150 mm, mesh size 2 × 2 mm) and closed with a rubber band. Eclosing flies were collected manually and kept in transparent polypropylene cages (390 × 280 × 280 mm) with a net (fiberglass; 200 × 300 mm, mesh size 2 × 2 mm) integrated into the lid of each cage. Cages were illuminated with LED panels (Y51515227 184210, Barthelme, Nuremberg, Germany) in a light:dark photoperiod of 16:8 hours [24]. Eggs were collected in corrugated plastic cardboards fixed on wet sponges (see below). Every other day, eggs were collected on pine humus in black plastic boxes and sprayed with tap water to maintain constant humidity until hatching.

### Experimental design

**Fig 1:**
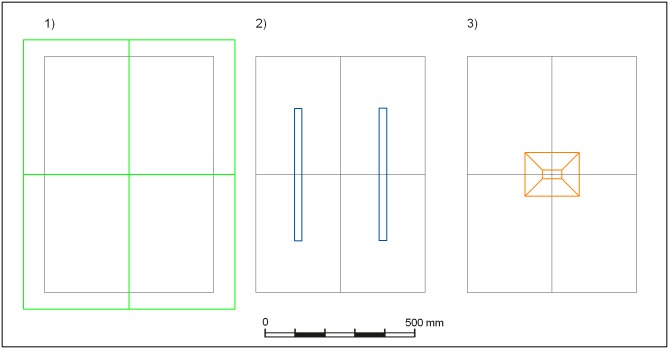
Top view of the position of the three light sources: 1) light-emitting diode (LED, green); 2) fluorescent lamp (FL, blue); and 3) halogen lamp (HL, amber); the contours of polyethylene cages are drawn in black.

Three artificial light sources were tested for oviposition and half-life of *H*. *illucens*. These were 1) four LED panels sized 445 × 350 mm consisting of 21 rows of LED strips with 12 LEDs per strip (Y51515227 184210, Barthelme, Nuremberg, Germany); 2) two parallel mounted FL sized 300 × 438 mm (4014501041710, Narva, Brand-Erbisdorf, Germany); and 3) one HL (QVF135 HAL-TDS500W K BK CN, Philips Lighting, Jiading, China; see http://www.lighting.philips.com for a technical drawing) (Fig 1). The spectra of all light sources were measured with a USB2000 fibre-optic spectrometer (300–885 nm; Ocean Optics Inc., Florida, USA), and the luminous intensity was measured with a luxmeter (Skye, SKP215 PAR Quantum Sensor, Wales, UK). Four replicate cages per light source were used, and the experiment was repeated three times in summer 2015. The experiments were terminated on Day 15.

The four replicate cages per light source were placed in a vertical distance from the light sources that resulted in an equal light intensity of 59 μmol m^-2^ s^-1^ at half height of the cages (Fig 1), following Zhang et al. (2010) [22]. Environmental conditions were 27 °C, 60% RH, and a 16:8 light:dark photoperiod, though under the HL, the temperature rose up to 29 °C when light was turned on. To avoid cross effects, light sources were separated with opaque walls. In Experiments 1 and 2, 40 male and 40 female flies aged < 24 hours were released per cage. Due to a breeding shortage, only 30 male and 30 female flies were used in Experiment 3. The position of the four cages under each light source was randomized daily after each data collection.

### Oviposition traits and adult fly half-life

Two batches of three layers of black corrugated plastic cardboard (40 × 30 mm) fixed on a wet irrigated cellulose sponge were offered as oviposition site and water source. Wet sponges were covered with 6 g chicken feedstuff mixed with tap water (60:40 w/v). Eggs were collected daily under a mosquito net to avert flies from escaping. To avoid exsiccation, egg clutches were placed in self-sealing bags immediately after collection. The egg clutches in the cardboards were counted and weighed using a Mettler Analytical Balance AE 166 Delta Range 0.1 mg (Mettler-Toledo Ltd., Ohio, USA). Further, the sponges, floor, wall, and lid of the cages were checked for eggs; these eggs were only weighed and not counted because frequently multiple egg clutches clustered together. After collecting the eggs, cardboards were replaced and sponges were washed and irrigated. Dead flies were collected and sexed daily, and the numbers of dead females and males were recorded. Based on these data, the pre-oviposition period, the total oviposition period, the average mass of eggs, and the half-life of flies were calculated.

### Statistical analyses

Data were computed in Excel 2010^®^ (Microsoft Corp., Redmond, USA), and graphs were established using SigmaPlot V13.0. (Systat Software Inc., San Jose, California, USA). One-way analysis of variance (ANOVA) was done to test differences of oviposition and half-life for each light source using SPSS 24 (IBM Corp., Armonk, New York, USA). To assess differences of fly survival among light sources, half-life was calculated separately for both sexes and independently from oviposition analyses.

## Results

**Fig 2:**
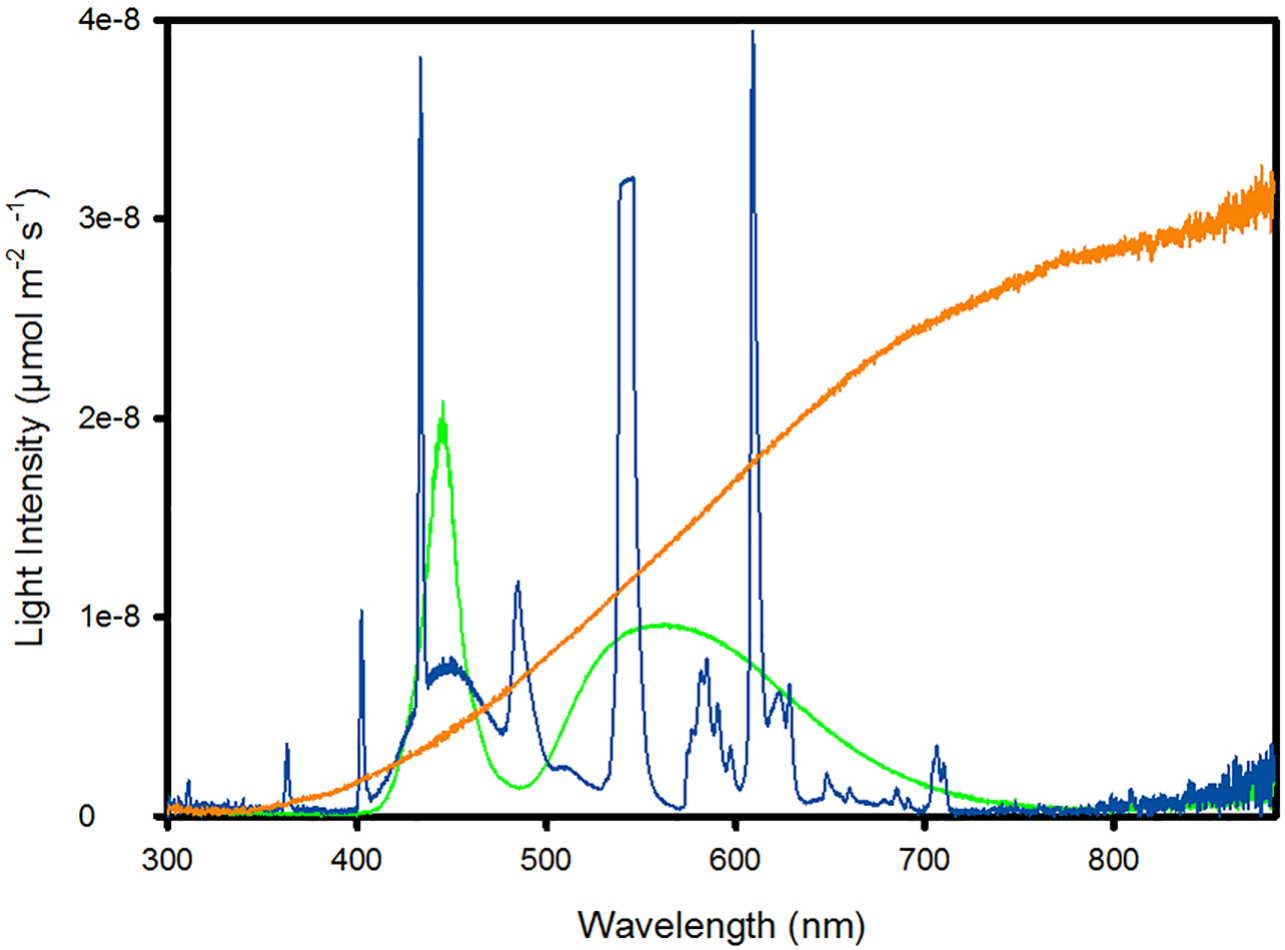
Light spectra (μmol m^-2^ s^-1^) of three artificial light sources: 1) light-emitting diode (LED, green); 2) fluorescent lamp (FL, blue); and 3) halogen lamp (HL, amber) at wavelengths from 300 to 885 nm.

Mating was observed, and oviposition was successful under all artificial light sources. The LED had a discontinuous spectrum in the range 400–750 nm with a peak found at 438 nm, the FL a multi-peaked and discontinuous spectrum from 311 to 711 nm with the highest intensities found at 430, 541, and 612 nm, and the HL a continuous spectrum beginning at 300 nm and with still increasing intensity at the longwave limit of the spectrometer’s range (Fig 2).

**Fig 3:**
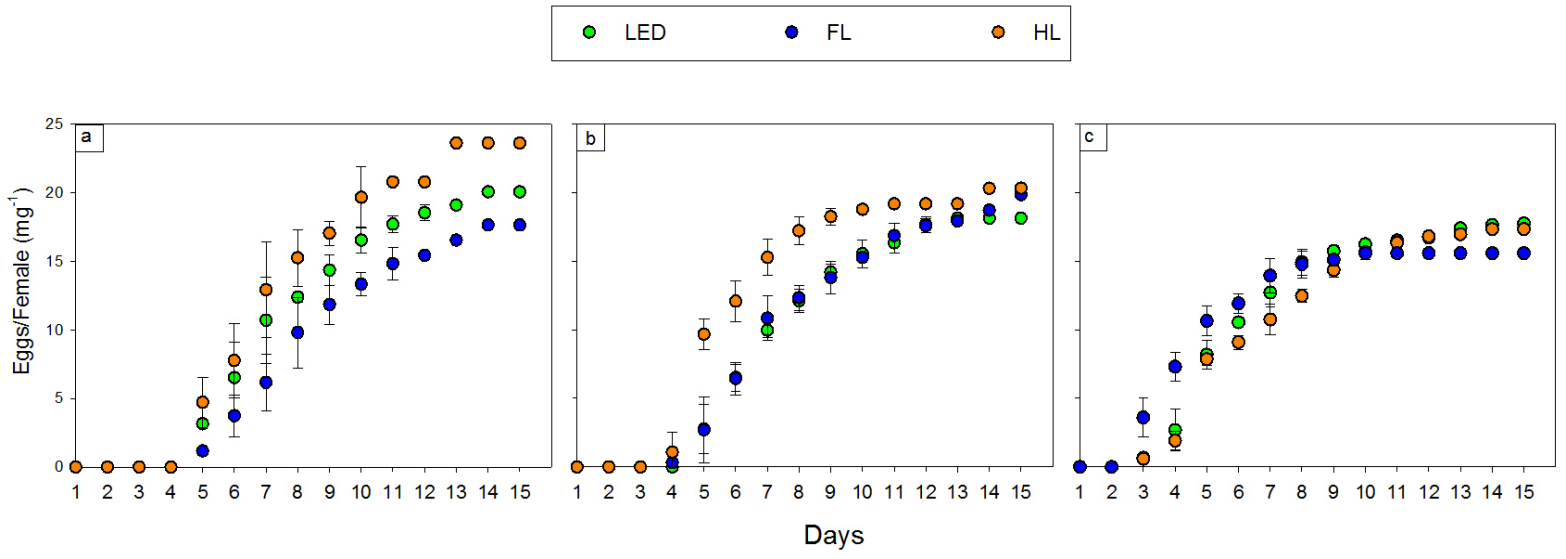
Accumulated egg mass per female (mg) and standard deviation for three artificial light sources: 1) light-emitting diode (LED, green); 2) fluorescent lamp (FL, blue); and 3) halogen lamp (HL, amber) during the 15 days for Experiment 1 [a], Experiment 2 [b], and Experiment 3 [c].

In Experiment 1, 2, and 3, only 22%, 10%, and 12% of the weighed eggs, respectively, were found in the cardboard. The remaining egg clusters were collected from sponges, walls, and lids of the cages. The pre-oviposition period ranged from two to four days. The oviposition period ranged from eight (HL in Experiment 1 and 2) to 13 days (LED in Experiment 3). The oviposition peak ranged from four to eight days, with an egg mass ranging from 3.7 to 8.6 mg. The average clutch mass ranged from 1.9 to 2.3 mg. The total egg mass ranged from 61.2 to 79.9 mg. In none of these five parameters, the three light sources differed significantly (Table 1, Fig 3).

**Table 1:** Selected life history traits of *Hermetia illucens* reared under three artificial light sources.

	Experiment 1				Experiment 2				Experiment 3			
	LED	FL	HL	*p*	LED	FL	HL	*p*	LED	FL	HL	*p*
**Pre-oviposition period (days)**	4	4	4	0.4	4	3	3	0.1	2	3	2	0.3
**Oviposition period (days)**	10	10	8	0.1	9	11	9	0.1	13	12	8	0.1
**Day of peak**	7	8	7	1.0	6	7	5	0.1	5	5	4	0.3
**Peak egg mass per female per day (mg)**†	4.2	3.7	5.2	0.1	3.8	4.4	8.6	0.1	5.5	5.9	3.7	0.1
**Average clutch mass (mg)**×	2.3	2.1	1.9	0.5	1.9	1.9	2.1	0.9	1.9	1.9	2.0	0.8
**Total egg mass per female (mg)**	64.8	61.3	79.9	0.6	68.9	69.8	75.4	1.0	65.9	64.5	61.2	1.0
**Male half-life (days)**•	13	11	7	**0.0**	>15҂	>15҂	12	**0.0**	>15҂	>15҂	11	**0.0**
**Female half-life (days)**•	11	6	4	**0.0**	13	14	9	**0.0**	13	12	9	**0.0**

LED = light-emitting diode; FL = fluorescent lamp; HL = halogen lamp; *p* = p-value (*p* < 0.05 = bold lettering)

^†^ Peak egg mass per female per day (mg) = the peak of egg mass weighed

^×^ Egg mass calculated using the number of egg clutches counted in the cardboards

^•^ Days until half-life was reached

^҂^ Experiment was terminated on Day 15, true half-life over 15 days and therefore unknown

**Fig 4:**
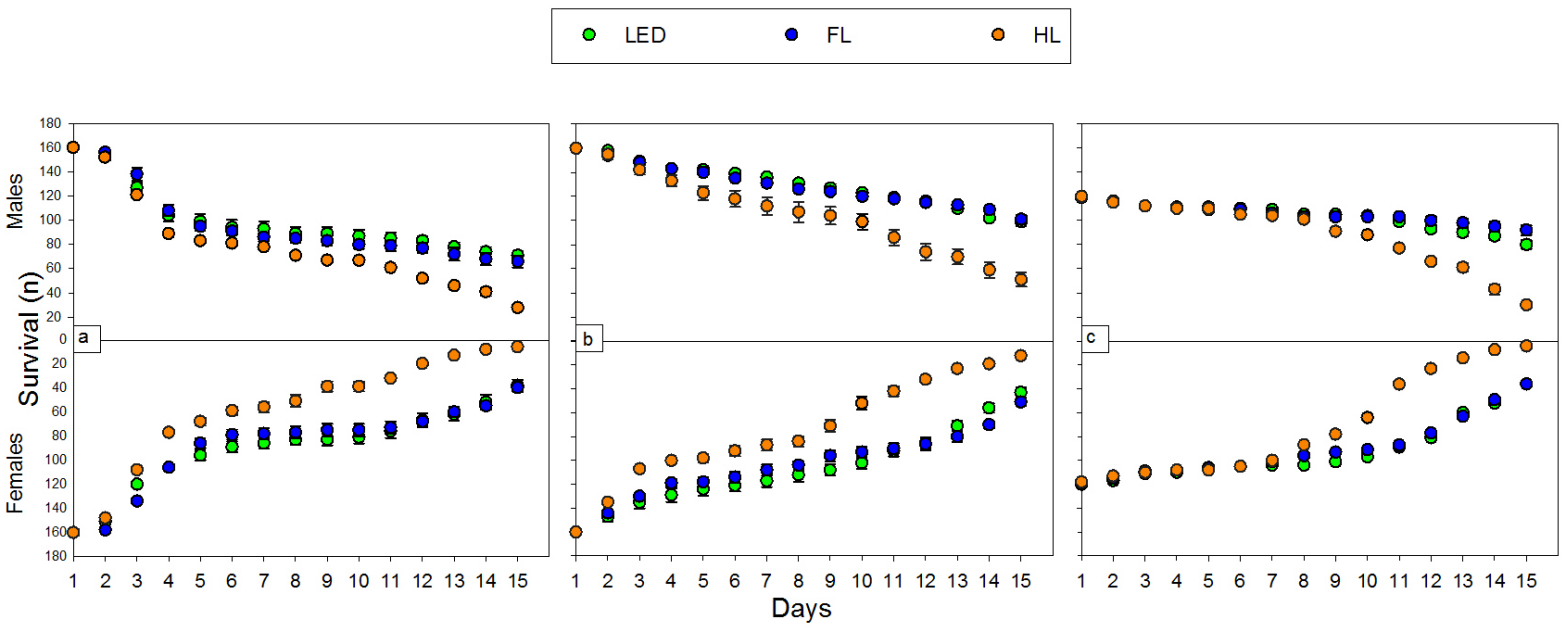
Male and female survival under three artificial light sources: 1) light-emitting diode (LED, green); 2) fluorescent lamp (FL, blue); and 3) halogen lamp (HL, amber) during the 15 days for Experiment 1 [a], Experiment 2 [b], and Experiment 3 [c].

Male and female half-life differed significantly among the light sources and throughout the experiments (Table 1, Fig 4). Male half-life ranged from seven to more than 15 days, and female half-life ranged from four to 13 days. HL caused significantly shorter half-life of males and females in all experiments. Generally, males lived longer, though the difference between male and female half-life was not significant over all light sources throughout all experiments (Table 1).

## Discussion

Black Soldier Fly males depend on sunlight to detect females entering their mating sites [20]. Interception occurs in flight, while copula occurs in descending to the ground or directly on the ground [20, 25, 26]. Most mating pairs can be observed under direct sunlight, while few mating pairs can be observed in winter or on cloudy days [20, 22]. In the past, many studies indicated that indoor rearing of BSF is challenging because the mating behavior is sensitive to space and light [20]. These studies normally used large cages ranging from 1800 × 1200 × 1500 mm to 2000 × 2000 × 4000 mm, and mating occurred under the influence of sunlight in greenhouses [17, 20, 23, 25]. However, Nakamura et al. (2016) showed that rearing BSF in small cages (270 × 270 × 270 mm) is possible, and several studies proved that rearing under artificial light is feasible [21–23]. For instance, mating and oviposition were successful under LED panels (400–800 nm, JLM-LTG20 W, Jinxing Rantoon Co. LTD., Zhejiang Province, China) [23] and quartz iodine lamps (350–2500 nm, QVF135, Philips Lighting Ltd., Eindhoven, Netherlands) [22]. In contrast, no mating was observed when using artificial light sources like the Pro Ultralight light system (Hydrofarm Inc., Petaluma, California, USA) [20], the Sylvania Gro Lux system (400–680 nm, Orson Sylvania Inc., Danvers, Massachusetts, USA) [20], or rare earth lamps (350–450 nm, Engineering University Infrared Technology Research Institute, Hei Lung Kiang, China) [17, 22]. The authors concluded that wavelengths from 332 to 535 nm influence mating behavior [21] and that the absence of those wavelengths might be the reason for mating failure and infertile eggs [20].

In this study, we obtained oviposition in small cages (390 × 280 × 280 mm) and under all three artificial light sources. The pre-oviposition period ranged from two to four days, which is similar to previous studies [23], and the difference among the three light sources was not significant over all experiments. The oviposition period ranged from eight to ten days; differences among light sources were not significant. Comparable results (7.6 and 9.4 days) were published by Nakamura et al. (2016) [23]. Oviposition peaked between four and seven days after emergence, similar to many studies where the peak in oviposition ranged from four to 17 days after emergence [20, 22, 23]. Zhang et al. (2010) reported an oviposition peak after 17 days under sunlight, while under quartz iodine lamps (350–2500 nm) oviposition peaked after 13 days [22]. Similar results were obtained by Oonincx et al. (2016) with an earlier oviposition peak at 10 days under fluorescent tubes (300–650 nm), while the oviposition peak under LED (365–515 nm) occurred after 16 days. The authors monitored the number of hatched larvae and asserted that the number was significantly higher for the LED treatment. Hence, they hypothesized that fertile eggs might be oviposited later [21]; in contrast, Tomberlin et al. (2002) reported an oviposition peak after four days of emergence under sunlight [20]. It is unclear if eggs oviposited earlier are infertile. Additional research should be conducted to understand if the time of oviposition is connected to the fertility of the eggs.

It is noteworthy that the BSF colony used in our experiments was reared under the same LED light source that was used for one of the experimental groups. Thus, it is possible that the flies underwent evolutionary adaptation to this specific light source in the generations preceding the experiments. The alternatives of using sunlight as control or a full design were impractical. Sunlight was impractical because of the latitudinal position of our laboratory (47.3° N). A full design, that is, establishing separate colonies for each light source and testing all possible combinations, would have tripled the experimental effort and would have been beyond the technical possibilities in our laboratory.

Nakamura et al. (2016) hypothesized that high density of adult flies is an important factor in achieving mating and oviposition, using 50 males and 50 females (0.0050 flies cm^-3^). In our study, we obtained oviposition using only 40 male and 40 female flies (0.0026 flies cm^-3^; Experiment 1 and 2) or 30 male and 30 female flies (0.0020 flies cm^-3^; Experiment 3), whereas Oonincx et al. (2016) obtained eggs using only 10 male and 10 female flies (0.0007 flies cm^-3^), demonstrating that a lower density of adult BSF does not negatively influence oviposition [21], at least over the range studied so far. We cannot completely exclude an effect of the different numbers of individuals in Experiment 1&2 versus Experiment 3; this difference affected both fly density in the cage and statistical power. However, we do not expect this influence to be severe because the number of individuals was of a similar magnitude. The density of flies influences the length of the oviposition period and the day at which oviposition peaks. For small-scale rearing and future studies concerning oviposition of BSF, it should be more accurately analyzed how the number of adults in relation to the volume of the cage influences oviposition.

The average clutch mass ranged from 1.9 to 2.3 mg and was constant over time. The peak egg mass per female per day and the total egg mass per female did not differ significantly across the three light sources for all experiments, indicating that oviposition behavior was comparable under all light sources. Tomberlin et al. (2002) defined an average clutch mass of 1.5 mg, obtained for egg clutches oviposited in a greenhouse under the influence of sunlight, though it is difficult to determine an egg clutch, as sometimes different females lay eggs clustered together [27]. It has even been observed that BSF females are attracted to ovipositing on conspecific eggs [28]. To the best of our knowledge, in previous studies concerning light influence on BSF mating and oviposition authors decided to count the number of egg clutches [19, 27]. Hence, a comparison across studies is difficult. Furthermore, it should be mentioned that numerous flies oviposited outside of the cardboards (>90% of egg clutches), preferentially in sponges but also on the floor, walls, and lids. In nature, eggs are deposited directly on or near a humid substrate of decomposing material [28], and perhaps females prefer to oviposit eggs on a humid material. However, using the cardboards is still a popular method to collect eggs. In the future, it should be considered to quantitatively compare the preference of female BSF to different materials already in use for egg collection, for instance humid paper towels, wet coco peat, and wet cellulose sponges [21, 23].

Half-life of male and female BSF differed significantly over all experiments and light sources. Especially for HL, half-life of flies was significantly shorter; this might be due to the higher temperature under HL (up to 29 °C) [23]. Studies showed that adult longevity and oviposition period are negatively correlated with increasing temperature [29]. The half-life of BSF males and females was significantly higher for LED. Males mostly survived longer than females, ranging from six to over 15 days for males and three to 13 days for females. However, the egg-laying peak occurred four days after emergence, meaning that the 50% surviving females were still very productive. Tomberlin et al. (2002) found oocytes were not present in female BSF after three days of oviposition and speculated that females mate and oviposit only once in life [20]. Although adult BSF do not feed [2], providing flies with sugar-water extended their life span to up to 73 days [21, 23]. However, if females indeed oviposit only once in their lifetime, adding sugar would unnecessarily increase breeding time and costs. Further studies are necessary to asses if introduction of sugar-water increases the number of eggs per female.

Although HL is commonly used for rearing BSF [30], we do not recommend this light source for small-scale rearing or rearing in incubators because of its higher energy consumption and increased heat production. Concerning oviposition, half-life, and energy efficiency [31], the LED showed the best results and can therefore be recommended for rearing BSF.

This study supports previous studies that show that small-scale indoor rearing of BSF under artificial light sources is possible. The results will be useful for designing a small-scale indoor rearing system for BSF. However, further studies will be needed on the influence of light source and oviposition site on egg-fertility to optimize BSF rearing under indoor conditions.
