# Correction: Influence of three artificial light sources on oviposition and half-life of the Black Soldier Fly, *Hermetia illucens* (Diptera: Stratiomyidae): Improving small-scale indoor rearing

**DOI:** 10.1371/journal.pone.0226670

**Published:** 2019-12-12

**Authors:** Carina D. Heussler, Andreas Walter, Hannes Oberkofler, Heribert Insam, Wolfgang Arthofer, Birgit C. Schlick-Steiner, Florian M. Steiner

The Data Availability statement is incorrect. The raw data underlying this study are not provided in the published paper. The authors have provided the data as Supporting Information files S1 Table and S2 Table.

The Statistical analyses subsection of the Material and methods is incomplete. The correct paragraph is: Data were computed in Excel 2010® (Microsoft Corp., Redmond, USA), and graphs were established using SigmaPlot V13.0. (Systat Software Inc., San Jose, California, USA). One-way analysis of variance (ANOVA) was done to test differences of oviposition, and one-way analysis of covariance (ANCOVA) was done to test differences of survival per day for each light source using SPSS 24 (IBM Corp., Armonk, New York, USA) and PAST 3.22 (https://folk.uio.no/ohammer/past/), respectively. When a significant difference (using an alpha of 0.05) was found in an ANOVA or ANCOVA analysis comparing all three light sources, pairwise comparisons of light sources were added, followed by Bonferroni correction for multiple comparison.

In light of this Correction, the first and third sentences of the third paragraph of the Results need to be updated. The correct paragraph is: Male and female survival differed significantly among the light sources and throughout the experiments (Table 1, Fig 4, S1 Table). Male half-life ranged from seven to more than 15 days, and female half-life ranged from four to 13 days. HL caused significantly shorter survival of males and females in all experiments (S2 Table). Generally, males lived longer, though the difference between male and female half-life was not significant over all light sources throughout all experiments (Table 1).

**Table 1:** Selected life history traits of *Hermetia illucens* reared under three artificial light sources.

	Experiment 1				Experiment 2				Experiment 3			
	LED	FL	HL	*p*	LED	FL	HL	*p*	LED	FL	HL	*p*
**Pre-oviposition period (days)**	4	4	4	0.4	4	3	3	0.1	2	3	2	0.3
**Oviposition period (days)**	10	10	8	0.1	9	11	9	0.1	13	12	8	0.1
**Day of peak**	7	8	7	1.0	6	7	5	0.1	5	5	4	0.3
**Peak** **egg mass per female per day (mg)**†	4.2	3.7	5.2	0.1	3.8	4.4	8.6	0.1	5.5	5.9	3.7	0.1
**Average clutch mass (mg)×**	2.3	2.1	1.9	0.5	1.9	1.9	2.1	0.9	1.9	1.9	2.0	0.8
**Total egg mass per female (mg)**	64.8	62.1	79.9	0.6	70.5	69.8	75.4	1.0	65.9	64.5	61.2	1.0
**Male half-life (days)•**	13	11	7	**0.0**	>15҂	>15҂	12	**0.0**	>15҂	>15҂	11	**0.0**
**Female half-life (days)•**	11	6	4	**0.0**	13	14	9	**0.0**	13	12	9	**0.0**

LED = light-emitting diode; FL = fluorescent lamp; HL = halogen lamp; *p* = p-value (*p* < 0.05 = bold lettering)

^†^Peak egg mass per female per day (mg) = the peak of egg mass weighed

^×^ Egg mass calculated using the number of egg clutches counted in the cardboards

^•^ Days until half-life was reached; *p*-value based on the number of surviving BSF per day

^҂^ Experiment was terminated on Day 15, true half-life over 15 days and therefore unknown

In Table 1, the values for Total egg mass per female (mg) in the FL column of Experiment 1 and LED column of Experiment 2 are incorrect. The correct values are 62.1 and 70.5, respectively. In addition, there is an error in the third footnote for Table 1. The correct footnote is: Days until half-life was reached; *p*-value based on the number of surviving BSF per day. Please see the correct Table 1 here.

## Supporting information

S1 Table Number (n) of surviving male and female Black Soldier Flies per day under the influence of three artificial light sources: 1) light-emitting diode (LED); 2) fluorescent lamp (FL); and 3) halogen lamp (HL) during the 15 days for Experiment 1, Experiment 2, and Experiment 3. (DOCX)

S2 Table Pairwise one-way ANCOVAs for pairwise comparisons of survival of male and female Black Soldier Flies per day under the influence of three artificial light sources: 1) light-emitting diode (LED); 2) fluorescent lamp (FL); and 3) halogen lamp (HL) during the 15 days for Experiment 1, Experiment 2, and Experiment 3, followed by Bonferroni corrections for multiple comparison (* = p-Value < α 0.0167). (DOCX)
