# Supplementary material for: Correction: Influence of three artificial light sources on oviposition and half-life of the Black Soldier Fly, Hermetia illucens (Diptera: Stratiomyidae): Improving small-scale indoor rearing
Source: PLoS One. 2019 Dec 12;14(12):e0226670. doi: 10.1371/journal.pone.0226670 (PMC6907789; doi:10.1371/journal.pone.0226670)
Supplement: S1 Table — (DOCX) [file pone.0226670.s001.docx]

**S1 Table. Number (n) of surviving male and female Black Soldier Flies per day under the influence of three artificial light sources: 1) light-emitting diode (LED); 2) fluorescent lamp (FL); and 3) halogen lamp (HL) during the 15 days for Experiment 1, Experiment 2, and Experiment 3.**

| **Experiment 1** | **Days** | **n of surviving males** | | | **n of surviving females** | | |
| --- | --- | --- | --- | --- | --- | --- | --- |
|  |  | **LED** | **FL** | **HL** | **LED** | **FL** | **HL** |
|  | 1 | 160 | 160 | 160 | 160 | 160 | 160 |
|  | 2 | 152 | 156 | 152 | 151 | 158 | 148 |
|  | 3 | 127 | 138 | 121 | 120 | 134 | 108 |
|  | 4 | 104 | 108 | 89 | 106 | 106 | 77 |
|  | 5 | 99 | 95 | 83 | 96 | 86 | 68 |
|  | 6 | 94 | 91 | 81 | 89 | 79 | 59 |
|  | 7 | 93 | 86 | 78 | 86 | 78 | 56 |
|  | 8 | 92 | 85 | 71 | 83 | 77 | 51 |
|  | 9 | 89 | 84 | 68 | 83 | 75 | 39 |
|  | 10 | 87 | 81 | 67 | 81 | 75 | 39 |
|  | 11 | 85 | 80 | 61 | 76 | 73 | 32 |
|  | 12 | 83 | 78 | 52 | 67 | 68 | 20 |
|  | 13 | 78 | 73 | 46 | 62 | 60 | 13 |
|  | 14 | 74 | 69 | 41 | 52 | 55 | 8 |
|  | 15 | 71 | 67 | 30 | 38 | 40 | 6 |
| **Experiment 2** | 1 | 160 | 160 | 160 | 160 | 160 | 160 |
|  | 2 | 158 | 154 | 155 | 147 | 144 | 135 |
|  | 3 | 149 | 148 | 142 | 135 | 130 | 107 |
|  | 4 | 143 | 143 | 133 | 129 | 119 | 100 |
|  | 5 | 142 | 140 | 123 | 124 | 118 | 98 |
|  | 6 | 139 | 135 | 118 | 121 | 114 | 92 |
|  | 7 | 136 | 131 | 112 | 117 | 108 | 87 |
|  | 8 | 131 | 126 | 107 | 112 | 104 | 84 |
|  | 9 | 127 | 124 | 104 | 108 | 96 | 71 |
|  | 10 | 123 | 120 | 99 | 102 | 93 | 52 |
|  | 11 | 119 | 118 | 86 | 92 | 90 | 42 |
|  | 12 | 116 | 115 | 74 | 86 | 86 | 32 |
|  | 13 | 110 | 113 | 70 | 71 | 80 | 23 |
|  | 14 | 102 | 109 | 59 | 56 | 70 | 19 |
|  | 15 | 99 | 101 | 51 | 43 | 51 | 12 |
| **Experiment 3** | 1 | 120 | 120 | 120 | 120 | 120 | 120 |
|  | 2 | 119 | 120 | 120 | 120 | 119 | 118 |
|  | 3 | 116 | 116 | 115 | 117 | 115 | 113 |
|  | 4 | 112 | 112 | 112 | 111 | 109 | 110 |
|  | 5 | 111 | 110 | 110 | 110 | 108 | 108 |
|  | 6 | 111 | 109 | 110 | 107 | 106 | 108 |
|  | 7 | 110 | 109 | 105 | 105 | 105 | 105 |
|  | 8 | 109 | 106 | 104 | 104 | 101 | 100 |
|  | 9 | 105 | 103 | 101 | 104 | 96 | 87 |
|  | 10 | 105 | 103 | 91 | 101 | 93 | 78 |
|  | 11 | 104 | 103 | 88 | 97 | 91 | 64 |
|  | 12 | 99 | 103 | 77 | 89 | 87 | 36 |
|  | 13 | 93 | 100 | 66 | 81 | 77 | 23 |
|  | 14 | 90 | 98 | 61 | 60 | 63 | 14 |
|  | 15 | 87 | 95 | 43 | 52 | 49 | 7 |
