# Supplementary material for: Correction: Influence of three artificial light sources on oviposition and half-life of the Black Soldier Fly, Hermetia illucens (Diptera: Stratiomyidae): Improving small-scale indoor rearing
Source: PLoS One. 2019 Dec 12;14(12):e0226670. doi: 10.1371/journal.pone.0226670 (PMC6907789; doi:10.1371/journal.pone.0226670)
Supplement: S2 Table — (DOCX) [file pone.0226670.s002.docx]

**S2 Table. Pairwise one-way ANCOVAs for pairwise comparisons of survival of male and female Black Soldier Flies per day under the influence of three artificial light sources: 1) light-emitting diode (LED); 2) fluorescent lamp (FL); and 3) halogen lamp (HL) during the 15 days for Experiment 1, Experiment 2, and Experiment 3, followed by Bonferroni corrections for multiple comparison (*= p-Value < α 0.0167).**

| **Experiment 1** | | | | |
| --- | --- | --- | --- | --- |
|  | **Males** | | **Females** | |
| **Pair of Lights** | **p-Value** | **Significance after Bonferroni correction** | **p-Value** | **Significance after Bonferroni correction** |
| **LED vs FL** | 0.623 |  | 0.730 |  |
| **LED vs HL** | 0.001 | * | 0.000 | * |
| **FL vs HL** | 0.004 | * | 0.000 | * |
| **Experiment 2** | | | | |
|  | **Males** | | **Females** | |
| **Pair of Lights** | **p-Value** | **Significance after Bonferroni correction** | **p-Value** | **Significance after Bonferroni correction** |
| **LED vs FL** | 0.182 |  | 0.313 |  |
| **LED vs HL** | 0.000 | * | 0.000 | * |
| **FL vs HL** | 0.000 | * | 0.000 | * |
| **Experiment 3** | | | | |
|  | **Males** | | **Females** | |
| **Pair of Lights** | **p-Value** | **Significance after Bonferroni correction** | **p-Value** | **Significance after Bonferroni correction** |
| **LED vs FL** | 0.247 |  | 0.371 |  |
| **LED vs HL** | 0.000 | * | 0.000 | * |
| **FL vs HL** | 0.000 | * | 0.000 | * |
